# New Biocidal Additive for Resin-Based Dental Composites: Is Modification with Didodecyldimethylammonium Bromide (DDAB) Effective?

**DOI:** 10.3390/polym18141792

**Published:** 2026-07-22

**Authors:** Maja Zalega, Witold Jakubowski, Joanna Nowak, Kinga Bociong

**Affiliations:** 1 Department of General Dentistry, Medical University of Lodz, Pomorska 251, 92-213 Lodz, Poland; maja.zalega@stud.umed.lodz.pl; 2 Division of Biophysics, Institute of Materials Science and Engineering, Lodz University of Technology, Stefanowskiego 1/15, 90-924 Lodz, Poland; witold.jakubowski@p.lodz.pl; 3 University Laboratory of Materials Research, Medical University of Lodz, Pomorska 251, 92-213 Lodz, Poland; joanna.nowak.1@umed.lodz.pl

**Keywords:** dental composite, antibacterial composite, antibacterial activity, quaternary ammonium salt, mechanical properties, antimicrobial properties, shrinkage stress

## Abstract

The study aimed to develop and preliminarily characterize experimental resin-based dental composites (RBCs). In addition to the composites, didodecyldimethylammonium bromide (DDAB) was used as a biocide in various concentrations (0–3 wt%). Hardness (HV), flexural strength (FS), and modulus of elasticity, as well as diametral tensile strength (DTS) of composites, were examined. Additionally, shrinkage stress, surface free energy (SFE), water sorption (Wsp), and solubility (Wsl) were determined. Cytometric analysis, including biocidal surface testing and susceptibility to colonization by *Streptococcus mutans, Escherichia coli*, and *Candida albicans*, assessed antibacterial activity. The HV of RBCs varied from 27.2 ± 1.5 to 31.5 ± 1.8 depending on DDAB amount, FS—67.0 ± 16.1 MPa for control composite and 79.2 ± 14.5 MPa for modified composite. All DTS values exceed 24 MPa. Shrinkage stress is highest for the composite with 0.25 wt% DDAB—18.5 ± 1.9 MPa—and lowest for the composite with 1 wt% DDAB—15.0 ± 2.5 MPa. Wsp is lowest in the control group (37.19 ± 1.69 µg/mm^3^) and highest for 1 wt% modifier (42.11 ± 2.75 µg/mm^3^). Wsl was at its lowest in control group (1.98 ± 1.58 µg/mm^3^), and highest for modification with 1 wt% (3.91 ± 1.74 µg/mm^3^). For RBCs modified with 3 wt% DDAB after 60 min, an increase in dead cells is observed: 51% for *Escherichia coli*, 71% for *Streptococcus mutans*, and 24% for *Candida albicans*. Preliminary results show that DDAB effectively reduced the presence of the tested pathogens and contraction stress; however, at certain concentrations it negatively influences hardness and water sorption of composites. The presented findings highlight both the potential and the limitations of DDAB-modified RBCs and underline the need for further studies, including cytotoxicity and genotoxicity assessments, release analyses and evaluation of composites’ ageing behavior.

## 1. Introduction

The development of modern resin-based composites (RBCs) focuses on improving their properties. It is essential to achieve improved mechanical properties and enhance aesthetics. Research is also aimed at modifying RBCs to obtain biostatic or biocidal properties that promote oral health. Composites are among the most used materials in dentistry [1]. With their help, we can create a wide range of tooth reconstructions—direct and indirect. RBCs have many advantages. Their disadvantages include polymerisation shrinkage, polymerisation contraction stress, and susceptibility to surface plaque accumulation [2]. Polymerisation shrinkage occurs during the curing of the polymeric phase of the dental composite. Then, a reduction in the material’s volume and the formation of marginal leaks and fissures are observed. This phenomenon creates space for bacterial invasion and biofilm formation [3,4]. The bacteria most associated with tooth decay are *Streptococcus mutans* and related groups, including *Streptococcus salivarius*, *Streptococcus parasanguinis*, and *Veillonella parvula*. *Candida albicans* and *Candida dubliniensis* fungi are also strongly associated with tooth decay [5,6]. Biofilm formation on the surface of composite restorations is one cause of secondary caries. The problem of secondary caries is significant, reaching almost 60% of cases [7]. It is the most common cause of dental filling failure and appears to be a major clinical challenge associated with RBCs. Secondary caries leads also to the material’s chemical and mechanical degradation in the long run. It is necessary to perform a new filling/reconstruction, which involves a renewed loss of the patient’s tissues and incurs costs [8,9,10].

One of the most well-known resin matrix biocidal modifiers are quaternary ammonium compounds (QACs) [11,12]. Examples of these modifications include the methacrylate-based monomers enriched with quaternary ammonium, i.e., dimethyl-hexadecyl-methacryloxyethyl-ammonium iodide (DHMAI) [12,13], methacryloyloxydodecylpyridinium bromide (MDPB) [13,14], dimethylaminododecyl methacrylate (DMADDM) [15], or quaternary urethane-dimethacrylate ammonium derivatives QAUDMA-m [16,17]. Another interesting option is to use quaternary ammonium salts (QASs) [18]. QASs are characterised by a strong biocidal effect. Their action leads to the destruction of the pathogen’s cell membrane, denaturing enzymes and proteins, and disrupting cellular metabolism [11,19,20]. These agents are used as disinfectants due to their antimicrobial properties. QASs are also used in the cosmetics industry as emollients and surfactants [21]. They are also used as preservatives in cosmetic and food products [21,22]. To achieve biocidal properties, Yao et al. used modified microcapsule nanoparticles containing an antibacterial inorganic filler (NIF) with a quaternary ammonium salt [23], and Huang et al. added the positively charged quaternary amine N^+^ to MAE-HB. This reduced pathogen adhesion by modulating the expression of glucosyltransferases, thereby limiting biofilm formation and the occurrence of secondary caries [24].

**Figure 1:**
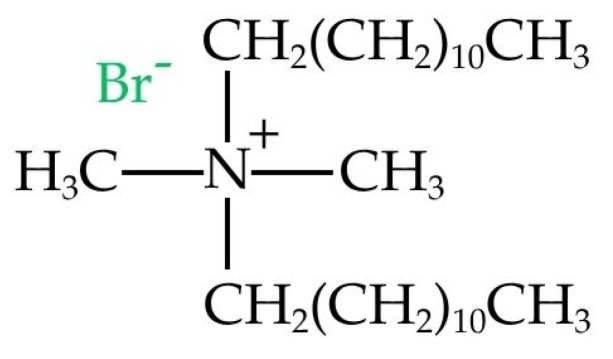
Structure of didodecyldimethylammonium bromide (DDAB).

Didodecyldimethylammonium bromide (DDAB), Figure 1, a cationic surfactant, is one of the examples of QASs. It is characterized by its ability to reduce surface tension [25]. DDAB is used for drug transport via nanostructured lipid carriers (NLCs), and has been proposed for systemic delivery of lipophilic compounds. DDAB-coated NLCs have been shown to reduce the viability of human glioblastoma multiforme cells [26]. The substance is used as a disinfectant; Kamarov et al. used the salts against SARS-CoV-2 [27].

The rationale for selecting DDAB in the present study is based on both its established antimicrobial mechanism and its structural similarity to other QASs previously investigated in dental materials. Studies on the DDAB analogue, dimethyldioctadecylammonium bromide (DODAB), have shown that such modifiers can impart antimicrobial properties without inducing cytotoxic or genotoxic effects [28]. Additionally, there is a lack of data regarding the incorporation of DDAB into polymer-based dental composites and its influence on their physicochemical and mechanical properties. To address this gap, DDAB was selected as a potential RCBs biocidal modifier.

The hypothesis of this work is that the use of DDAB will improve the properties of the experimental RBCs, while also reducing the amounts of pathogens.

## 2. Materials and Methods

### 2.1. Materials

**Figure 2:**
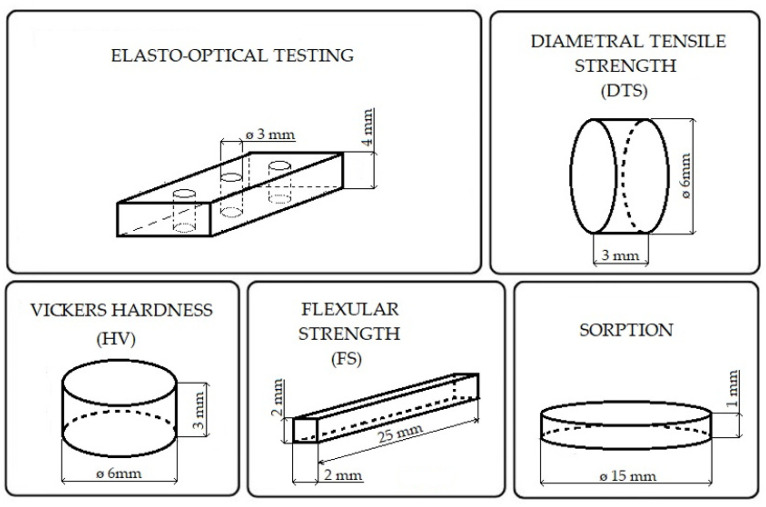
Schemes of samples used in tests.

Monomers—bisphenol A glycerol dimethacrylate (bis-GMA, purity ≥97%), diurethane dimethacrylate (UDMA, purity ≥97%), triethylene glycol dimethacrylate (TEGDMA, purity ≥95%), 2-hydroxyethyl methacrylate (HEMA, purity ≥97%) (all from Sigma-Aldrich, St. Louis, MO, USA)—were used to make the matrix in a ratio of 40/40/10/10% by weight. As part of the photoinitiator system, camphoroquinone (CQ, purity ≥97%) in the amount of 0.4 wt%, polymerization process stabilizer butylated hydroxytoluene (BHT, purity ≥99%) in the amount of 0.1 wt%, and co-initiator 2-(dimethylamino)ethyl methacrylate (DMAEMA, purity ≥98%) in the amount of 0.9 wt% were used. In the next step, the matrix was filled. For this purpose, Arsil silica (Zakłady Chemiczne Rudniki S.A., Rudniki, Poland) silanized with 3-methacryloxypropyltri-methoxysilane (γ-MPTS, Unisil Sp. z o.o., Tarnów, Poland) was used at 45 wt%. Didodecyldimethylammonium bromide (DDAB, purity 98%) (Sigma-Aldrich, St. Louis, MO, USA) was used to modify the material in amounts of 0.25, 0.5, 1.0, 2.0, and 3.0 wt% (relative to the weight of the resin matrix). Depending on the test type, silicone moulds were prepared to fabricate samples (Figure 2). The composite was placed in the moulds, and the top layers were protected with tape (Hawe Striproll, Kerr, Bioggo, Switzerland), which prevents the formation of an inhibition layer. The moulds were covered with laboratory slides, and in the next step, they were exposed for a time of 20 s, using THE CURE TC-01 polymerisation lamp (SPRING; Norristown, PA, USA) with a power of 1200 mW/cm^2^ at a material thickness of 1.5 mm. The fibre-optic tip was in close contact with the surface being irradiated. To ensure stable lamp power, the radiometer system (DigitalLight Meter 200 from Rolence Enterprice Inc., Taoyuan, Taiwan) was used before and after photopolymerization.

Before testing, all samples were stored in distilled water at 37 °C.

### 2.2. Methods

While studying the effect of the modification, Vickers hardness, diametral tensile strength, and flexural strength were examined. Shrinkage stresses generated during the photopolymerization of the composite were also determined.

#### 2.2.1. Hardness

A semi-automatic ZHμ-2 hardness tester (Zwick/Roell, Ulm, Germany) with a load of 9.81 N was used to determine Vickers hardness (HV). Three specimens were prepared for each group, each a cylinder with a height of 3 mm and a diameter of 6 mm. On each specimen, three indentations were made (*n* = 9).

#### 2.2.2. Diametral Tensile Strength

DTS was determined using nine specimens (identical to HV specimens, *n* = 9). During the test, the specimens were subjected to compression along the diameter, perpendicular to the major axis, until destruction (sliding speed—2 mm/min).

#### 2.2.3. Flexural Strength

A three-point bending test (TPB) was used to determine the composite’s flexural strength (FS). The flexural modulus (Ef) was also determined during the test. The test was conducted following ISO 4049 [29]. Six specimens (*n* = 6, per the above standard, five specimens were required; one extra specimen was prepared to cover potential losses or visible defects) with dimensions of 25 × 2 × 2 mm were used for the test group. The crosshead’s sliding speed during the test was 1 mm/min.

#### 2.2.4. Shrinkage Stresses

During the photopolymerization of the composite, shrinkage stresses are generated. An elasto-optical method using a Gunt FL200 circular polariscope (Gunt Gerätebau GmbH, Barsbüttel, Germany) was used to determine them. Stress values were calculated using transformed Timoshenko and elastic theory formulas (a detailed description of the calculation method was presented in our previous publications [30,31,32,33]). For each composite, three measurements (*n* = 3) were made.

#### 2.2.5. Surface Free Energy

The surface free energy (SFE) of the materials was also determined during the study. It was calculated from wetting angle measurements, and the method of Owens and Wendt. A pair of polar and non-polar liquids (diiodomethane and water) was used. When testing the composites, 7 drops of each liquid (*n* = 7) were used. The surface free energy is the sum of the dispersion components (γd) and the polar components (γp). The process was described in an article by Szczesio-Wlodarczyk et al. [31].

#### 2.2.6. Microbiological Test

The antibacterial properties of DDAB-modified experimental RBCs were also investigated. Cytometric analyses—biocidal analysis of surfaces and analysis of susceptibility to colonization against *Escherichia coli, Streptococcus mutans*, and *Candida albicans*—were performed. For surface activity analysis, we used a typical “contact-killing” setup: a cell suspension of each pathogen was applied directly to the top surface of flat samples arranged horizontally in Petri dishes, without immersing them in the bulk suspension. After incubation (at 37 °C for 10 or 60 min), the bacterial suspension was carefully collected from the surface and then subjected to the staining procedure associated with the ‘Viability/Cytotoxicity Assay kit for Bacteria Live and Dead Cells’ (ImmuniQ, Żory, Poland). After incubation with fluorescent reagents, the results were read using an Accuri C6 flow cytofluorimeter (BD Biosciencem, San Jose, CA, USA) and then analysed using BD CSampler software (BD Biosciencem). Simultaneous standardization was performed using a positive control (live cells) and negative control (dead cells). In all, 20,000 passages were collected each time, corresponding to 20,000 cells analysed. A suspension collected after 24 h of culture (logarithmic growth phase culture) was used—in the case of *Escherichia coli* strain DH5α, LB medium with composition: NaCl (1%), bactopeptone (1%) and yeast extract (0.5%) and pH equal 7.0. For *Candida albicans*, a YPG medium was used with the composition: yeast extract (1%), bactopeptone (1%), and glucose (2%), and the pH was 7.4. For *Streptoccocus mutans* an MSB medium (mitis-salivarius-bacitracin from BD Biosciencem) was used [34].

For analysis of susceptibility to colonization the pairs of each sample type were cleaned by thorough rinsing with distilled water. Afterwards, samples were sterilized by immersion in 90% ethanol and then immersed in sterile deionized water. The samples thus prepared were placed in a medium suitable for the species tested—LB medium for *Escherichia coli*, MSB medium for *Streptococcus mutans* and YPG medium for *Candida albicans*. Each pair of samples was associated with two independent replicates. A standardised number of cells was introduced into 200 mL of culture medium prepared in this way: the accepted standard is 1 mL of culture in the stationary growth phase with an absorbance at 680 nm equal to 1, which corresponds to approximately 2 × 10^3^ bacterial or yeast cells. The culture was conducted for 24 h at 37 °C. At the end of the incubation, the samples were removed in a sterile manner and rinsed with sterile distilled water to remove unadhered cells. The surface of the sample was subjected to a bacterial or yeast cell counting procedure using a fluorescence microscope. The method is based on Live/Death staining with simultaneous use of two fluorescent dyes, bis-benzimidine and propidium iodide [35]. The first one penetrates inside the bacteria/yeast and intercalates with the DNA, resulting in UV-stimulated luminescence, allowing visualization of live cells. The second also attaches to the DNA, but does not penetrate the cell membrane, resulting in the visualization of dead cells. The study was carried out on a GX71 inverted-optics fluorescence microscope equipped with a DP73 digital camera (Olympus, Tokyo, Japan). A minimum of five images were taken for each sample at a random location (but reasonably proximate to the centre of the sample).

The tests were conducted according to protocols described in detail in the article by Nowak et al. [36]. *Escherichia coli* was chosen as an example of Gram-negative bacteria and *Streptococcus mutans* as a Gram-positive bacterium, and a trigger of caries.

#### 2.2.7. Absorption and Solubility

To determine the absorbency of the materials, samples were prepared using a silicone mould with a diameter of 15 mm and a width of 1 mm. The material was placed in the moulds and irradiated with THE CURE TC-01 polymerisation lamp (SPRING; Norristown, PA, USA) with a power of 1200 mW/cm^2^ at a material thickness of 1.5 mm. Absorption and solubility tests were performed in accordance with ISO 4049 [29]; hence, five samples (*n* = 5) were prepared for each experimental composite. The prepared samples were placed in a vacuum desiccator (Duran^®^, Mainz, Germany) and dried to a constant weight (m_1_), and then immersed in distilled water (37 °C, 7 days). After 7 days, the samples were weighed and then dried again to a constant weight (m_3_). Weight measurements were performed using a RADWAG AS 160/C/2 balance, Radom, Poland. Based on the differences in weight, the water sorption (Wsp) and solubility (Wsl) ratios were calculated:

$$Wsp=\frac{m_{2}-m_{3}}{V}\times 100$$

$$Wsl=\frac{m_{1}-m_{3}}{V}\times 100$$

m_1_—Sample mass after conditioning before immersion [µg];
m_2_—Mass of the sample after immersion in water for 7 days [µg];
m_3_—Sample mass after reconditioning [µg].

After obtaining the results of the above-described tests, statistical analysis was performed using Statistica v.13 (Tibco Software Inc., Krakow, Poland). Arithmetic means, standard deviations, maxima and minima, and medians were calculated. The Shapiro–Wilk test was used to assess the distribution’s normality. The Kruskal–Wallis test was used to test the hypotheses when the data did not follow a normal distribution; otherwise, the Scheffe test was applied. In the next step, the analysis determined which groups were statistically different from the others; for this purpose, multiple comparisons of the mean ranks of all groups were used, according to the assumption of an alpha level of significance of 0.05.

## 3. Results

The results obtained in this study are gathered in Table 1 and Table 2.

### 3.1. Hardness

The highest hardness after DDAB modification was obtained for the composite with 0.5 wt% (31.5 ± 1.8), while the lowest was observed for the composite enriched with 3.0 wt% salt (27.2 ± 1.5). The Shapiro–Wilk test showed inconsistency with a normal distribution. This was true for measurements of material modified with 0.5% DDAB-modified weights (*p* < 0.05). The Kruskal–Wallis test showed statistically significant differences between the C0 and C2.0 (*p* = 0.021503)/C3.0 (*p* < 0.001); C0.25 and C2.0 (*p* = 0.009625)/C3.0 (*p* < 0.001); C0.5 and C2.0 (*p* < 0.001)/C3.0 (*p* < 0.001); and C1.0 and C3.0 (*p* < 0.001) (each number near C—concentration, indicates amount of DDAB in wt%).

### 3.2. Diametral Tensile Strength

In the case of DTS, the highest values were observed for the 0.25 wt% DDAB composite modification (36.3 ± 3.7 MPa), while the lowest were observed for the control material (29.3 ± 5.1 MPa). The Shapiro–Wilk test showed no inconsistency with a normal distribution. An analysis of variance was performed, which showed inconsistencies. The Scheffe test was performed, which showed significant statistical differences between the DTS of the control material and the material modified with 0.25 (*p* = 0.049811) and 0.5 wt% DDAB (*p* = 0.045902).

### 3.3. Flexural Strength

The flexural strength test showed that the highest values were achieved by the composite material enriched with 1.0 wt% DDAB (79.2 ± 14.5 MPa), while the lowest value was achieved by the control material (67.0 ± 16.1 MPa). Statistical analysis performed using the Shapiro–Wilk test showed inconsistency with a normal distribution. Kruskal–Wallis tests showed no statistically significant differences between unmodified and DDAB-modified composite material.

### 3.4. Flexural Modulus

The modulus of elasticity of the composite material reaches its highest values for the material modified with 1.0 wt% DDAB (4030.0 ± 219.0 MPa), while it is the lowest for the control material (3701.7 ± 398.3 MPa). The Shapiro–Wilk test showed no inconsistency, with a normal distribution. An analysis of variance was performed, which showed inconsistencies. Scheffe’s Test was performed, which showed no statistically significant differences between the values of the modulus of elasticity of the control composite and the sub-modified materials.

### 3.5. The Shrinkage Stress

The highest shrinkage stress is observed for the control composite, while the lowest values are obtained by composites with the addition of 1 wt% of the modifier (Table 2).

### 3.6. Surface Free Energy

The highest SFE was obtained for the control composite, at a concentration of 2.0 wt% (43.4 ± 4.1 mJ/m^2^) or 3.0 wt% (43.4 ± 6.1 mJ/m^2^) DDAB. The lowest, on the other hand, was for the modification with 1.0 wt% DDAB (35.3 ± 8 mJ/m^2^; Table 3).

### 3.7. Water Sorption and Solubility

The lowest sorption and solubility were obtained for the control group—37.19 ± 1.69 µg/mm^3^ and 1.98 ± 1.58 µg/mm^3^ (Table 4). On the other hand, the highest sorption and solubility values were obtained by material enriched with 1 wt% DDAB (42.70 ± 1.80 µg/mm^3^, 3.91 ± 1.74 µg/mm^3^). No statistically significant differences were observed between the groups.

### 3.8. Cytometric Analysis—Biocidal Analysis of Surfaces

**Figure A1:**
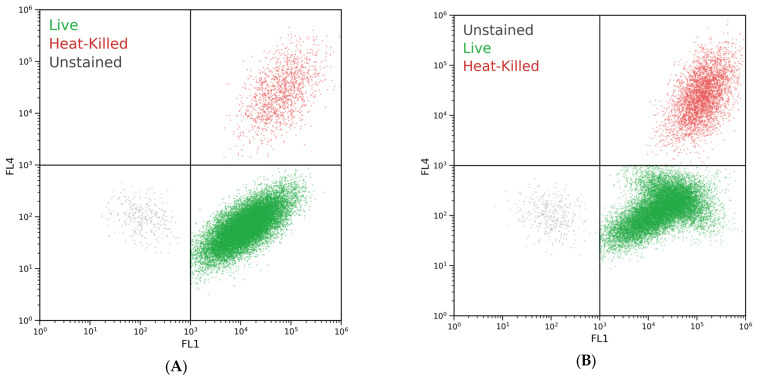

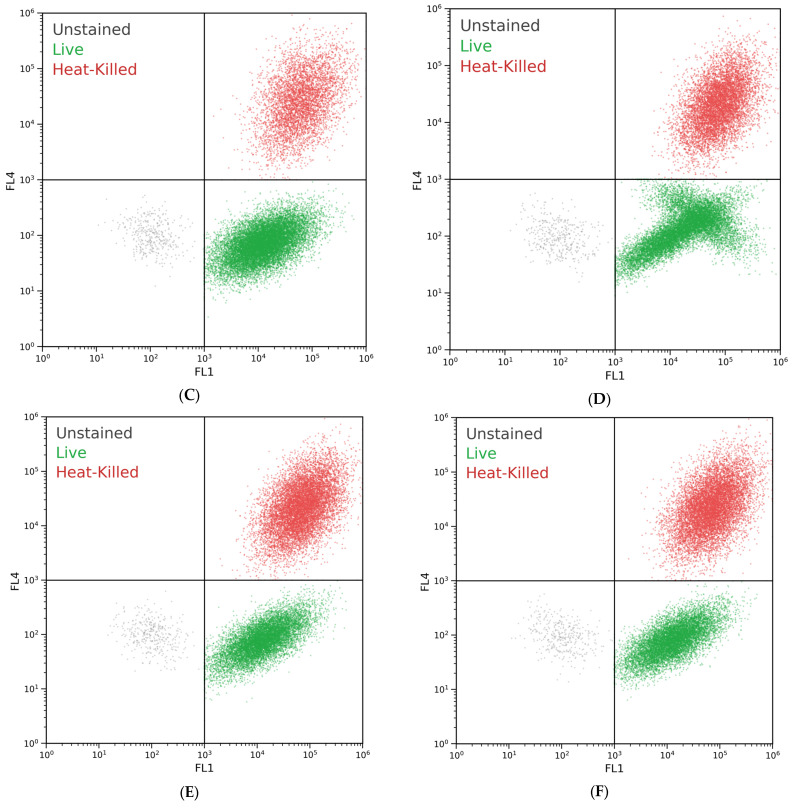
Cytometric plots of composites against *Escherichia coli*: (**A**)—control, (**B**)—composite with 0.25 wt% of DDAB, (**C**)—composite with 0.5 wt% of DDAB, (**D**)—composite with 1 wt% of DDAB, (**E**)—composite with 2 wt% of DDAB, and (**F**)—composite with 3 wt% of DDAB.

**Figure A2:**
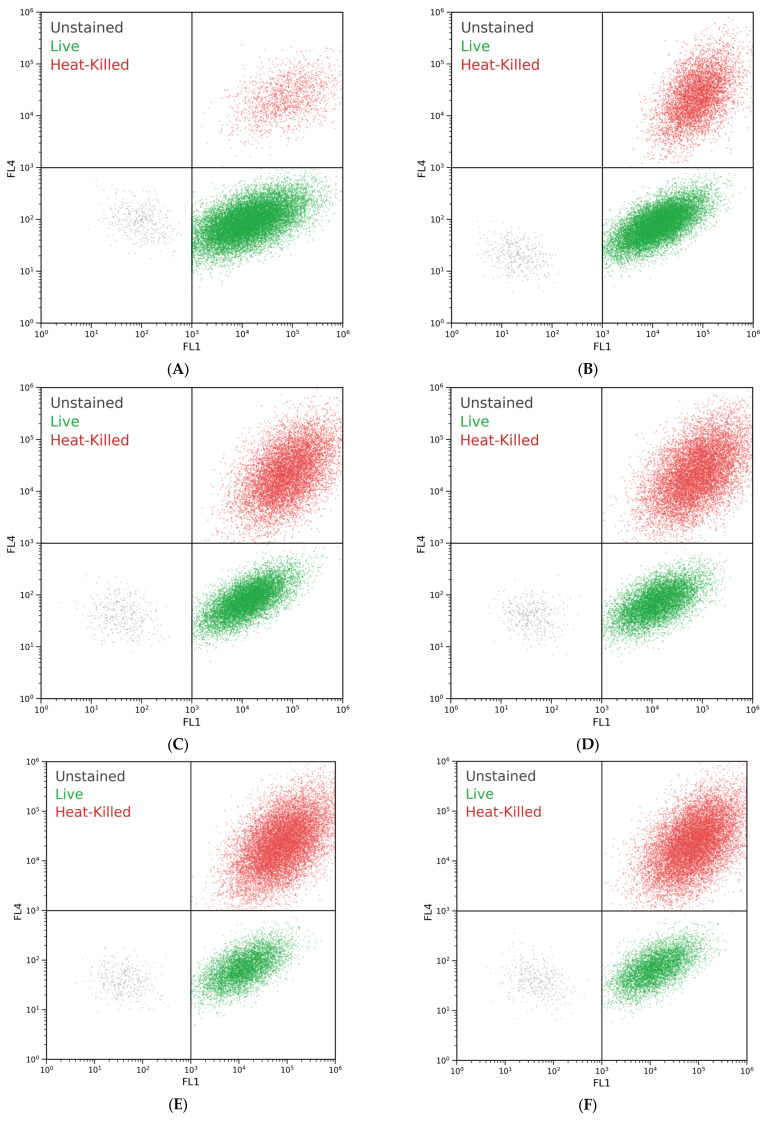
Cytometric plots of composites against *Streptococcus mutans*: (**A**)—control, (**B**)—composite with 0.25 wt% of DDAB, (**C**)—composite with 0.5 wt% of DDAB, (**D**)—composite with 1 wt% of DDAB, (**E**)—composite with 2 wt% of DDAB, and (**F**)—composite with 3 wt% of DDAB.

**Figure A3:**
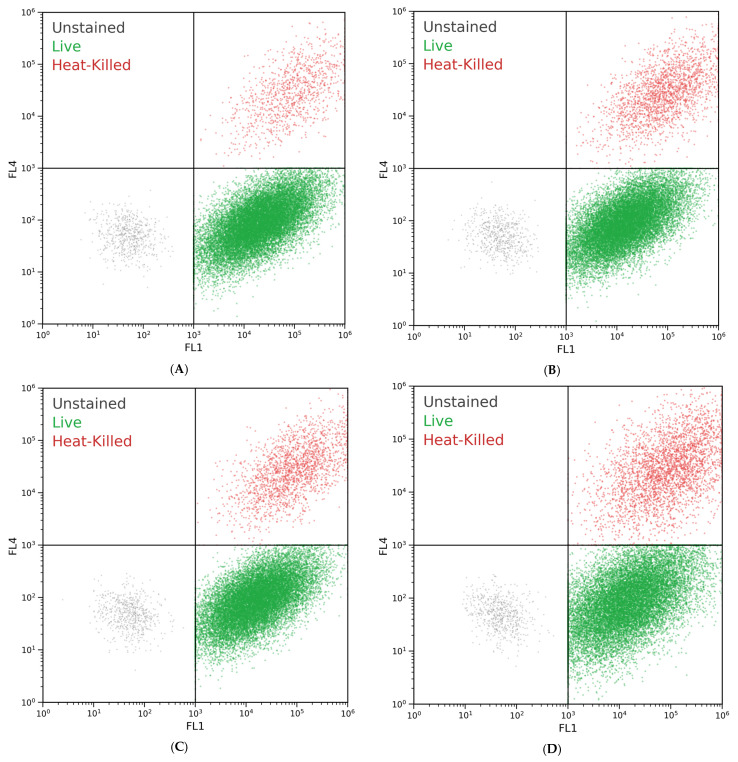

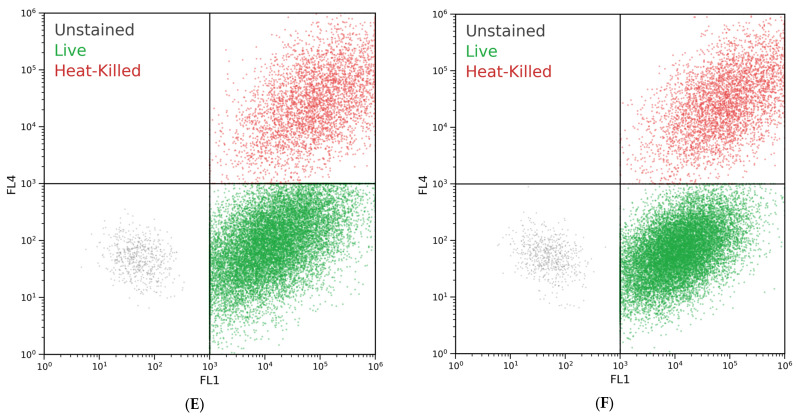
Cytometric plots of composites against *Candida albicans*: (**A**)—control, (**B**)—composite with 0.25 wt% of DDAB, (**C**)—composite with 0.5 wt% of DDAB, (**D**)—composite with 1 wt% of DDAB, (**E**)—composite with 2 wt% of DDAB, and (**F**)—composite with 3 wt% of DDAB.

For surface biocidal and antifungal activity against *Streptococcus mutans*, the highest number of dead cells was 70.59% (3 wt% DDAB, 60 min). In the case of *Candida albicans*, even with 3 wt% DDAB and a 60 min treatment, only 23.77% of the cells were observed to be dead (Table 5, Table 6 and Table 7, Figure A1, Figure A2 and Figure A3).

### 3.9. Analysis of Susceptibility to Colonization

**Figure 3:**
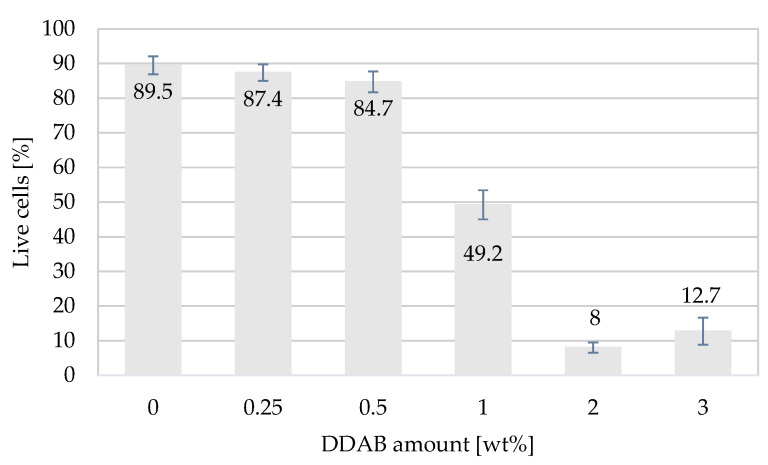
Analysis of susceptibility to colonization by *Escherichia coli*.

**Figure 4:**
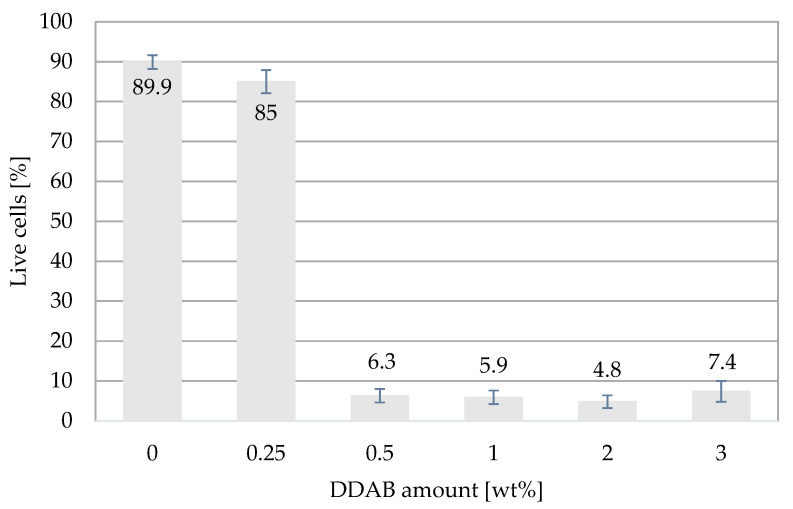
Analysis of susceptibility to colonization by *Streptococcus mutans*.

**Figure 5:**
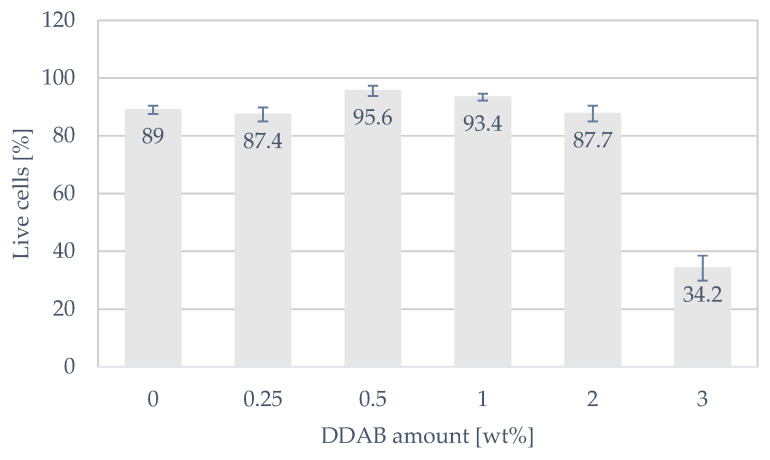
Analysis of susceptibility to colonization by *Candida albicans*.

Analysis of susceptibility to colonization showed that 2 wt% DDAB has the most effective effect against Streptococcus mutans (live cells—4.8 ± 1.6%). Despite the use of the highest concentration of DDAB, the effect against Candida albicans is the lowest (live cells: 34.2 ± 4.3%). The results are collected in Figure 3, Figure 4 and Figure 5.

## 4. Discussion

We assumed that a prepared experimental resin-based composite, in addition to possessing satisfactory mechanical properties not worse than those of the control composite, would exhibit bactericidal properties. Analysis of the data obtained during the study allows us to indicate the benefits and drawbacks of the proposed modification. It should be noted that the quaternary salts are well known for their antibacterial properties [11,18,37,38]. To the best of our knowledge, this is the first study using didodecyldimethylammonium bromide (DDAB) in dental composite composition.

It was noted that the highest hardness was achieved for the composite modified with 0.5 wt% DDAB (HV = 31.5 ± 1.8). Similar values were also observed for other experimental composites [39,40,41,42,43,44,45] and even for some commercial restorative materials [44,46]. The analysis of the available literature indicated that, for commercial materials, the minimum hardness ranged from 40 to 50. This value is dictated by the need to achieve hardness close to the HV ranges for dentin (approx. 65) and enamel (approx. 275) [45,47]. The noticeable hardness of our composites decreases significantly only at higher modifier concentrations, whereas at lower concentrations, there are no differences compared to the control material without DDAB. The decrease in hardness may be due to a change in the organic-to-inorganic phase ratio resulting from the incorporation of an organic additive. A similar relationship is observed for diametral tensile strength: the highest value at 0.5 wt% DDAB and the lowest at 2 and 3 wt%. Still the DTS of modified samples is higher than the control and higher than the minimum permissible value for this type of material. The presence of DDAB, due to its structure and hydrophilicity [36] can plasticize the material, probably resulting in better tensile properties. A downward trend was also observed in flexural strength, but no statistically significant differences were observed. As the concentration of the modifier increased, the strength decreased [39]. Although there are no statistical differences between the FS values of modified and unmodified composites, there is a pronounced difference in strength values in favour of DDAB-modified materials. The highest value was achieved for experimental RBC incorporating 1.0 wt% DDAB (FS = 79.2 ± 14.5 MPa). The value achieved almost meets the criterion for polymer dental composites for chewing surfaces (80 MPa) and meets the strength requirements for less-stressed dental surfaces (50 MPa) [29]. In the case of the elastic modulus, no drastic fluctuations were observed, and the highest value was achieved for the composite also modified with 1.0 wt% DDAB (Ef = 4030.0 ± 219.0 MPa).

Another property determining the durability of RBCs in oral conditions is shrinkage stress. It occurs during material exposure—too high a polymerization shrinkage can negatively affect the mechanical properties of the composite and compromise the effective bonding of materials [4,48,49]. Based on the findings of our study, it follows that incorporation of 1 wt% or more of DDAB to the experimental composite leads to visible stress reduction. The explanation for this phenomenon may be some degree of composite plasticization (as evidenced by higher DTS values and lower HV values with increasing amounts of added salt), which theoretically allows greater mobility of macromolecules before reaching the gel point and, as a result, partial stress relaxation. This is very promising since a decrease in stress is accompanied by a rather invariable relaxation of the necessary mechanical properties.

However, attention should be paid to water sorption (W_sp_) and solubility (W_sl_) ratios. Unfortunately, water sorption of modified composites is near to or higher than the maximum value defined by the ISO 4049 standard [29]. This increases the risk of a deterioration in RBC properties over time and may compromise hydrolytic stability, dimensional stability, and overall long-term material durability. It should therefore be considered as a limitation of the tested formulation. Water solubility values for the experimental composites are similar to those achieved by commercial materials [50,51]. In compositions of this type, the aim is low solubility and low water absorbance, because this provides dimensional stability for the reconstruction and limits swelling. There is then a lower risk of discoloration, and the material is chemically stable, which increases its biocompatibility [52,53].

The results show that modification with DDAB at low concentrations leads to a slight decrease in SFE, especially for composites with 0.25–1 wt% additive. There is a positive correlation between SFE and bacterial adhesion; hence, lowering SFE may reduce bacterial adhesion, leading to lower susceptibility to biofilm formation and secondary caries [54,55,56]. Such a surface will be more resistant to hydrophilic bacteria, for example, *Streptococcus mutans*. Bilgili et al. and Zhang et al. demonstrate that obtaining lower SFE values allows for the reduction of bacterial biofilm formation [56,57]. The length of the alkyl chain of the modifier used should also be considered. Nadagouda et al. suggest that the best biocidal properties are achieved with 14–16 carbon chains [58]. The DDAB chain is shorter than DODAB, yet a noticeable biocidal effect is still evident. Based on the research conducted, it was found that DDAB is effective against *Streptococcus mutans*, even at low concentrations. It has also been shown that higher concentrations of the modifier are necessary to achieve antifungal efficacy. During the biocidal analysis of surfaces, two exposure times were used: 10 and 60 min. During preparation, dental tubes are not 100% cleaned; trace amounts of pathogens remain [59,60]. The 10 min exposure comprises the early phase of adsorption and the initial action of the biocide, while the 60 min exposure is intended to simulate the long-term effects. These incubation times align with the literature on the rapid action of quaternary ammonium surfactants, where bactericidal effects are observed within 5–60 min due to pathogen membrane destabilization [20]. Selection of these points enables demonstration of time-dependent cell mortality increase (Table 5, Table 6 and Table 7, Figure A1, Figure A2 and Figure A3), confirming DDAB modification efficacy without requiring longer incubations (unlike the colonization test, which used 24 h). The experimental setup used, as well as the lack of visible turbidity in the medium, suggests that the dominant mechanism is contact killing resulting from DDAB surface modification, rather than mass release of the biocide into the solution volume. Compared with the Nowak study, which focused on composites with longer-chain QAS, composites with DDAB have slightly greater antifungal and biocidal effectiveness against *Candida albicans* and *Streptococcus mutans* [36]. Similarly to the above-mentioned studies, we noticed a negative correlation between increasing amounts of DDAB in experimental RBCs and the viability of microorganisms. *Streptococcus mutans* is proven to be the most susceptible pathogen, whereas *Candida albicans* displays higher resistance to quaternary ammonium salts. It should be noted that susceptibility to colonization is also reduced, and the materials exhibit biostatic properties [61]. *Candida albicans* resistance to DDAB may be due to the structure of its cell wall, which is thicker and more complex, making it more difficult for DDAB molecules to penetrate it [62]. Furthermore, biofilms involving *Candida albicans* are associated with increased resistance to biocides because cells within the biofilm are surrounded by a self-produced extracellular matrix [63,64]. Nevertheless, the primary objective of the presented study was to reduce bacterial colonisation associated with secondary caries; therefore, the observed predominance of antibacterial activity over antifungal activity is beneficial, while still leaving room for further optimisation of the formulation to enhance its antifungal activity in future studies.

Given the findings reported herein, the initial research hypothesis was partially confirmed: DDAB can be regarded as a promising biocide; nevertheless, the present composite formulation remains suboptimal and requires further concentration optimization and comprehensive physicochemical and biological validation.

The presented studies are part of a larger project focused on the modification of composites with DODAB analogues. In the coming stages, publications will be released regarding ongoing research into the cytotoxicity and resistance of these materials to accelerated ageing. In further studies, we will also focus on the analysis of DDAB release and the degrees of conversion of the composites.

A key limitation of the DDAB-modification is the observed increase in the composite’s water sorption compared to the control (e.g., from 37.19 ± 1.69 µg/mm^3^ for the control to 42.70 ± 1.80 µg/mm^3^ for the composite with 1 wt.% DDAB), while the ISO 4049 standard limit is 40 µg/mm^3^. Additionally, the material’s antifungal activity against *Candida albicans* was relatively low (e.g., only 25–35% reduction in viable cells at 60 min, vs >50% for bacteria), which may be a limitation. At elevated DDAB concentrations, the hardness of composites decreases (compared to control) which raises concerns regarding mechanical performance. These trade-offs highlight the need for further study and composition optimization. This work did not unequivocally determine whether the antibacterial activity of DDAB-containing resin-based composites results from contact-killing or from the release of DDAB, an issue that should be addressed in future research.

## 5. Conclusions

This preliminary study indicates that the incorporation of DDAB affects hardness and DTS but does not influence the flexural strength or flexural modulus of the prepared RBCs. The water sorption of experimental RBCs is compromised by the addition of DDAB, which is a major concern connected with the presented formulations. A correlation is observed between increased DDAB concentration and decreased contraction stress. The low concentrations of DDAB lead to a slight decrease in SFE—especially for composites with 0.25–1 wt% additive. There is also a strict dependence between time and the amount of DDAB and the viability of pathogens. In the case of biocidal and antifungal activity, the best efficacy was achieved with a modification of 3 wt% additive. The colonization susceptibility analysis showed that 2 wt% DDAB is most effective against bacteria, and 3 wt% against *Candida albicans*.

A composite containing 2 wt% DDAB is characterized by aspects demonstrating a technologically sound compromise among the concentrations and properties evaluated in this study.

## Figures and Tables

**Table 1:** Hardness (HV), diametral tensile strength (DTS), flexural strength (FS), and flexural modulus (E_f_) of modified composites.

DDAB Amount [wt%]	HV [-]	DTS [MPa]	FS [MPa]	E_f_ [MPa]
0	30.3 ± 1.8	29.3 ± 5.1	67.0 ± 16.1	3701.7 ± 398.3
0.25	30.5 ± 2.0	36.3 ± 3.7	78.3 ±10.2	3925.0 ± 282.0
0.5	31.5 ± 1.8	36.4 ± 4.3	75.8 ± 4.3	3900.0 ± 79.2
1.0	30.0 ± 1.9	35.5 ± 3.4	79.2 ± 14.5	4030.0 ± 219.0
2.0	28.8 ± 1.7	34.3 ± 6.0	76.6 ± 9.1	3971.7 ± 155.1
3.0	27.2 ± 1.5	33.2 ± 5.5	74.3 ± 9.8	3871.7 ± 290.3

**Table 2:** Effect of DDAB on shrinkage stress formed during resin-based composite polymerization (with the CURE lamp with output power 1200 mW/cm^2^ for 20 s per 1.5 mm of material).

DDAB Amount [wt%]	σ_r_ [MPa]	σ_ϴ_ [MPa]	σ_int_ [MPa]
0	8.1 ± 1.4	−10.4 ± 0.5	18.5 ± 1.9
0.25	8.9 ± 0.5	−10.5 ± 0.5	19.4 ± 0.9
0.5	8.3 ± 0.6	−9.9 ± 0.7	18.2 ± 1.2
1.0	6.7 ± 1.3	−8.3 ± 1.2	15.0 ± 2.5
2.0	7.2 ± 0.6	−8.6 ± 0.5	15.8 ± 1.1
3.0	7.5 ± 1.4	−8.9 ± 1.4	16.4 ± 2.8

σ_r_—radial stress, σ_θ_—circumferential stress, σ_int_—reduced shrinkage stress.

**Table 3:** Effect of DDAB salt on surface free energy.

**DDAB Amount [wt%]**	**Water Contact Angle [°]**	**Surface Free Energy [mJ/m^2^]**
0	66.27 ± 2.32	43.4 ± 6.1
0.25	74.05 ± 2.59	42.4 ± 7.2
0.5	77.68 ± 2.71	41.5 ± 8.7
1.0	67.49 ± 2.35	35.3 ± 8.0
2.0	70.57 ± 2.46	43.4 ± 4.1
3.0	66.27 ± 2.31	43.4 ± 6.1

**Table 4:** Effect of DDAB salt on sorption and solubility.

DDAB Amount [wt%]	Sorption (µg/mm^3^)	Solubility (µg/mm^3^)
0	37.19 ± 1.69	1.98 ± 1.58
0.25	41.65 ± 2.39	3.40 ± 1.91
0.5	40.76 ± 3.45	2.47 ± 0.53
1.0	42.70 ± 1.80	3.91 ± 1.74
2.0	39.86 ± 4.22	3.11 ± 0.58
3.0	42.11 ± 2.75	3.47 ± 0.90

**Table 5:** Cytometric analysis—Biocidal analysis of surfaces, using *Escherichia coli*.

	DDAB Amount [wt%]	Live Cells [%]	Dead Cells [%]
10 min	0	86.29	13.71
	0.25	84.49	15.52
	0.5	78.29	21.71
	1.0	76.64	23.36
	2.0	72.36	27.65
	3.0	67.94	32.07
60 min	0	82.54	17.46
	0.25	79.38	20.62
	0.5	73.53	26.48
	1.0	61.74	38.26
	2.0	49.96	50.04
	3.0	49.41	50.60

**Table 6:** Cytometric analysis—Biocidal analysis of surfaces, using *Streptococcus mutans*.

	DDAB Amount [wt%]	Live Cells [%]	Dead Cells [%]
10 min	0	86.77	13.23
	0.25	81.04	18.97
	0.5	72.76	27.24
	1.0	71.15	28.85
	2.0	65.56	34.44
	3.0	60.65	39.36
60 min	0	84.41	15.59
	0.25	69.93	30.08
	0.5	49.94	50.07
	1.0	42.71	57.30
	2.0	29.87	70.14
	3.0	29.41	70.59

**Table 7:** Cytometric analysis—Biocidal analysis of surfaces, using *Candida albicans*.

	DDAB Amount [wt%]	Live Cells [%]	Dead Cells [%]
10 min	0	93.27	6.74
	0.25	90.06	9.94
	0.5	89.27	10.74
	1.0	87.23	12.77
	2.0	82.76	17.24
	3.0	81.75	18.26
60 min	0	86.27	13.73
	0.25	89.96	10.05
	0.5	86.28	13.72
	1.0	87.05	12.96
	2.0	80.64	19.37
	3.0	76.24	23.77

## Data Availability

The data are available in a publicly accessible repository: https://doi.org/10.60941/gmq0-h511.
